# Prevalence of sexually transmitted infections (STIs) among first time visitors at STIs clinic in Hangzhou, China: Assessing the influence of the COVID‐19 pandemic

**DOI:** 10.1002/iid3.70009

**Published:** 2024-09-02

**Authors:** Jiyun Tian, Shi Chen, Xinzheng Li, Yong Teng, Baobing Chen

**Affiliations:** ^1^ Department of Clinical Laboratory The Third People's Hospital of Hangzhou Zhejiang China

**Keywords:** chlamydia trachomatis (CT), COVID‐19, HPV, HSV‐2, sexually transmitted infections (STIs), ureaplasma urealyticum (UU)

## Abstract

**Background:**

This study assesses the prevalence of sexually transmitted infections (STIs) in first time visitors to the STIs clinic in Hangzhou, China, considering different genders, ages and symptoms. And also explores howthe COVID‐19 pandemic has affected on STIs.

**Methods:**

From 2019 to 2023, 27,283 first time visitors were tested for nine distinct STIs, including *Human Papillomavirus* (HPV), *Human Immunodeficiency Virus* (HIV), syphilis, *Herpes Simplex Virus type 2* (HSV‐2), *Ureaplasma urealyticum* (UU), *Chlamydia trachomatis* (CT), *Neisseria gonorrhoeae* (NG), *Mycoplasma genitalium* (MG), and vaginal Candida.

**Results:**

Symptomatic male and female visitors showed overall STI‐positive rates of 39.27% and 59.20%, respectively(*p* < .001). The top three pathogens in both genders were HPV (47.56% and 56.71%), UU (29.21% and 56.47%), and HSV‐2 (22.41% and 52.94%). Among asymptomatic visitors, the total STI‐positive rate was 36.63% in males and 52.03% in females. Age‐stratified analysis revealed higher STI rates in visitors ≤ 20 or >50 years, regardless of gender and symptoms. During the COVID‐19 pandemic, symptomatic visitors showed lower positive rates for HPV, HIV, syphilis, and HSV‐2, while Candida, UU, CT, NG, and multiple infections increased. Among asymptomatic visitors, HPV had the lowest positive rate, while NG and multiple infections increased during the pandemic.

**Conclusion:**

STI prevalence is notably high, particularly in those aged ≤ 20 and >50 years. It emphasizes the need for enhanced health education, condom use, and vaccination. The COVID‐19 pandemic impacting STIs through varied factors, such as reduced sexual activity and clinical service interruption.

## 1 BACKGROUND

Sexually transmitted infections (STIs) constitute a category of infectious diseases transmitted through sexual, behavioral, and indirect contact. Common STIs encompass human immunodeficiency virus (HIV), syphilis, genital herpes, mycoplasma, chlamydia, gonorrhea, and genital candidiasis. The consequences of these infections can include sexual dysfunction, genital malformation, pelvic inflammatory diseases, cervical cancer, infertility, characteristic sequelae, spontaneous abortion, congenital malformation, premature birth, and other related conditions.^1^, ^2^ According to the *Global Progress Report on HIV, Viral Hepatitis and Sexually Transmitted Infections* published by the World Health Organization (WHO) in 2021, there are about 374 million patients newly diagnosed with STIs, of which 128.5 million patients newly diagnosed with CT (about 69.9 million males and 58.6 million females), and 82.4 million patients newly diagnosed with NG (about 35.9 million males and 46.49 million females) every year in the world. Among them, most patients are residents of developing countries. There are about 86 million patients newly diagnosed with STIs in the Western Pacific Region (including China), posing a severe challenge to public health.^3^

Epidemiological studies indicate that people living with STIs often exhibit co‐infections involving multiple pathogens. Zhong et al. highlighted that the positive rate of CT in people living with human papillomavirus (HPV) is notably higher than in those without HPV, irrespective of gender. Furthermore, the positive rate of CT coinfection is higher in people living with multiple HPV compared to those with single HPV, indicating a potential association between CT and HPV infections.^4^ Moreover, STIs such as HPV, NG, *Treponema pallidum*, and *Candida albicans* have been reported to heighten the susceptibility and transmission of HIV.^5^, ^6^, ^7^

Recent studies have highlighted the intricate relationship between STIs and host immune responses.^8^, ^9^ Chronic infections such as herpes simplex virus type 2 (HSV‐2) can modulate the immune system, leading to persistent inflammation and increased susceptibility to other infections.^10^ Conversely, immune responses can influence the natural history of STIs, affecting both the clearance of infections and the progression to disease. This bidirectional relationship underscores the importance of studying STIs within the broader context of immunity and inflammation.

The COVID‐19 pandemic has unavoidably disrupted the access to health care services. From the beginning of 2020, the stay‐at‐home order was implemented in China to keep an effective social distance among individuals. Despite this, health care professionals continued to work on the frontlines focusing on the prevention, control, treatment, and nursing of COVID‐19 patients. These professionals had to handle the shortage of experimental supplies and medical resources, which prevented patients from seeking timely diagnosis and treatment in the outpatient clinic.^11^, ^12^ It is possible that these factors combined may have led to a decrease in the screening, detection, and treatment of STIs. Furthermore, COVID‐19 itself has been shown to have significant effects on the immune system, often leading to immune dysregulation and chronic inflammation.^13^ For instance, in COVID‐19 patients, the prevalence and mortality rates of chronic obstructive pulmonary disease and tuberculosis have increased.^14^, ^15^ These changes in immune function during and after COVID‐19 infection could potentially alter susceptibility to STIs and the body's ability to manage these infections. However, the impact of COVID‐19 on STIs has not been further clarified by any scholars in the academic field.

Similar to many other developing countries, China faces a substantial burden of STIs. Due to mild or even absent symptoms and a potential sense of shame among some people living with STIs, seeking medical treatment in public health institutions may be limited. Consequently, there is scant information available regarding the incidence and prevalence of STIs, particularly with regard to multiple infections. In this study, various sexually transmitted pathogens were identified in first‐time visitors to the STIs clinic of Hangzhou Third People's Hospital, a prominent STIs clinic in major Chinese cities. Collected data spanned from January 1, 2019, to November 20, 2023. The evaluation process encompassed visitors of different genders, ages, and symptoms. Furthermore, the impact of the COVID‐19 pandemic on STIs was subject to analysis. By understanding how changes in social behavior and health care access during the pandemic have influenced STIs prevalence, we can better tailor interventions to reduce the burden of these infections. Additionally, our findings contribute to the broader understanding of the interplay between STIs, immunity, and inflammation, offering valuable insights for researchers and health care providers.

## 2 MATERIALS AND METHODS

### 2.1 Clinical data

From January 1, 2019, to November 20, 2023, comprehensive screening for STIs was conducted for all first time visitors to the STIs clinic of Hangzhou Third People's Hospital. The visitor cohort predominantly consisted of symptomatic individuals and asymptomatic individuals who underwent screening due to engaging in high‐risk sexual behavior or having contact with infected individuals. Symptomatic visitors were identified through self‐reported symptoms, such as dysuria, odynuria, itching, abnormal vaginal secretions, and abnormal vaginal bleeding. Additionally, people are classified as “symptomatic” if they consider themselves to be asymptomatic, but the clinicians detected abnormalities during physical examinations, including anogenital warts or herpes, urethral secretions in male visitors, and abnormal vaginal secretions, yellow purulent secretions at the cervical orifice, and cervical contact bleeding in female visitors. Visitors underwent one or more STI tests based on their specific conditions and economic considerations. This approach ensured a tailored and effective screening strategy for individuals presenting with diverse symptoms and risk factors. Therefore, not all visitors were tested for the whole pathogen profile, and some visitors only received partial tests.

For visitors who made multiple visits, their symptomatic status was based on their condition during the initial visit. Additionally, for the purpose of our analysis, we only included samples collected during the first visit for STI testing. Follow‐up samples were excluded to avoid including repeat testing from the same individual.

### 2.2 HPV testing

The HPV genotyping kit (PCR‐reverse dot blot hybridization assay) was purchased from Yaneng BIOscience Co., Ltd. (PRC). The kit can be applied to detect 23 HPV subtypes, including HPV 6, 11, 16, 18, 31, 33, 35, 39, 42, 43, 45, 51, 52, 53, 56, 58, 59, 66, 68, 73, 81, 82, and 83.

### 2.3 Ureaplasma urealyticum (UU), chlamydia trachomatis (CT), neisseria gonorrhoeae (NG), and mycoplasma genitalium (MG) testing

UU, CT, NG, and MG were detected using the Isothermal RNA Amplification assay kits purchased from Rendu Biotechnology Co., Ltd. The detection limit of the kits used during the procedure was 500 copies/mL.

### 2.4 HIV and HSV‐2 testing

The HIV and HSV‐2 antibody testing kits were purchased from Wantai Biopharm and Autobio Diagnostics Co., Ltd., respectively. These kits were applied to the detection of HIV and HSV‐2 antibodies in the serum of visitors by the enzyme‐linked immunosorbent assay (ELISA).

### 2.5 Syphilis testing

The syphilis antibody testing kit was purchased from Wantai Biopharm. The kit was applied to the detection of syphilis antibodies in the serum of visitors by ELISA. Visitors with a positive result were confirmed by the *Treponema pallidum* particle agglutination assay (TPPA).

### 2.6 Detection of Candida

0.5 ml of normal saline was added to the matching hose which containing vaginal secretions. After extrusion and mixing, the sample was coated on a glass slide and observed under an optical microscope to look for clue cells and the spores or hyphae of Candida.

### 2.7 Statistical analysis

SPSS 23.0 was used for the analysis of all collected data. The counting data were expressed as rates (%). Chi‐square tests were employed to evaluate the statistical significance of prevalence. However, when the sample size was small and did not meet the assumptions of the Chi‐square test, Fisher's exact test was used as an alternative to evaluate the statistical significance of prevalence. *p* < .05 was considered statistically significant.

## 3 RESULTS

### 3.1 Characteristics of the study participants

This study included 27,283 first time visitors to the STIs clinic of Hangzhou Third People's Hospital, from January 1, 2019, to November 20, 2023. The visitor cohort comprised 17,626 males and 9657 females, aged from 11 to 83 years. Of the total participants, 21,962 visitors exhibited symptoms, with 14,254 males and 7708 females. Additionally, 5321 visitors presented were asymptomatic, including 3327 males and 1949 females (Table 1).

**Table 1:** Prevalence of different STIs in different genders and symptoms.

	Symptomatic (21,962)				Asymptomatic (5321)				Overall (27,283)			
	Male (14,254)	Female (7708)	*X* ^2^	*p*	Male (3372)	Female (1949)	*X* ^2^	*p*	Symptomatic (21,962)	Asymptomatic (5321)	*X* ^2^	*p*
HPV	47.56%^a^ 1307/2748	56.71%^b^ 1412/2490	47.763	<.001	35.16% 405/1152	40.07% 232/579	3.999	.046	51.91% 2719/5238	36.80% 637/1731	118.973	<.001
HIV	0.9% 97/10779	0.9% 3/3162	22.248	<.001(F)	1.30% 32/2456	0.08% 1/1314	14.849	<.001 (F)	0.72% 100/13941	0.88% 33/3770	0.994	.319
syphilis	19.26% 2359/12248	38.41%^b^ 1903/4955	693.839	<.001	19.78% 498/2518	30.50% 438/1436	58.203	<.001	24.77% 4262/17203	23.67% 936/3954	2.109	.146
HSV‐2	22.41%^a^ 1044/4659	52.94%^b^ 703/1328	466.071	<.001	14.67% 131/893	36.83% 144/391	79.336	<.001	29.18% 1747/5987	21.42% 275/1284	31.733	<.001
UU	29.21% 837/2865	56.47% 589/1043	245.132	<.001	26.47% 248/937	61.44% 282/459	159.973	<.001	36.49% 1426/3908	37.97% 530/1396	0.963	.326
CT	12.46%^a^ 342/2745	9.01%^b^ 88/977	8.402	.004	4.76% 47/988	12.31% 64/520	28.484	<.001	11.55% 430/3722	7.36% 111/1508	20.337	<.001
NG	12.86%^a^ 338/2628	6.77% 70/1034	27.814	<.001	3.40% 32/942	7.35% 40/544	11.705	.001	11.14% 408/3662	4.85% 72/1486	49.564	<.001
MG	7.94%^a^ 216/2719	7.67% 66/861	0.07	.791	3.72% 28/752	10.34% 40/387	19.902	<.001	7.88% 282/3580	5.97% 68/1139	4.576	.032
overll	39.27%^a^ 5597/14254	59.20%^b^ 4563/7708	799.476	<.001	36.63% 1235/3372	52.03% 1014/1949	120.063	<.001	46.26% 10160/21962	42.27% 2249/5321	27.573	<.001
multiple infection	5.42% 773/14254	8.1%^b^ 624/7708	59.985	<.001	4.63% 156/3372	10.06% 196/1949	58.592	<.001	6.36% 1397/21962	6.62% 352/5321	0.462	.497

Abbreviations: CT, chlamydia trachomatis; HIV, human immunodeficiency virus; HPV, human papillomavirus; HSV‐2, herpes simplex virus type 2; MG, mycoplasma genitalium; NG, neisseria gonorrhoeae; STIs, sexually transmitted infections; UU, ureaplasma urealyticum.

a There was significant difference in the positive rate between symptomatic and asymptomatic male patients;

^b^ There was significant difference in the positive rate between symptomatic and asymptomatic female patients.

### 3.2 The prevalence of different STIs

Our results showed that the overall positive rate for STIs, defined as a positive result for at least one pathogen, was 46.26% (10,160/21,962) among all symptomatic visitors. More specifically, the positive rate among male visitors was 39.27% (5597/14,254), and among female visitors, it was 59.20% (4563/7708). This finding indicates a significantly lower positive rate of STIs in male visitors compared to female visitors (*p* < .001). The top three pathogens with the highest positive rates in both male and female visitors were HPV (47.56% and 56.71%), UU (29.21% and 56.47%), and HSV‐2 (22.41% and 52.94%). Among symptomatic visitors, the positive rates for HPV, syphilis, HSV‐2, UU, and multiple infections were higher in female visitors than in male visitors. However, the positive rates for HIV, CT, and NG were lower in female visitors compared to male visitors (Table 1).

Among asymptomatic visitors, the overall positive rate of STIs was 42.27% (2249/5321). The overall positive rate of STIs in male visitors was 36.63% (1235/3372), which was significantly lower than that in female visitors (52.03%, 1014/1949) (*p* < .001). The top three STIs with the highest positive rate in male visitors were HPV (35.16%), UU (26.47%), and syphilis (19.78%); while, those in female visitors were UU (61.44%), HPV (40.07%), and HSV (36.83%). Furthermore, considering the cohort of asymptomatic visitors, except for HIV, the positive rate of other STIs and multiple infections was significantly higher in female than in male visitors (Table 1).

In symptomatic male visitors, the overall positive rate of STIs and the positive rates of HPV, HSV‐2, CT, NG, and MG were significantly higher than in asymptomatic male visitors. However, there was no statistically significant difference in the positive rates of HIV, syphilis, UU, and multiple infections between symptomatic and asymptomatic male visitors. In symptomatic female visitors, the overall positive rate of STIs and the positive rates of HPV, syphilis, HSV‐2, CT, and Candida were higher compared to asymptomatic female visitors (Table 1).

### 3.3 STIs in visitors at different ages

The visitors included in this study were divided into five groups according to their age: <20, 21–30, 31–40, 41–50 and >50 years old. After that, the detection rate of STIs was counted. Among symptomatic male visitors, the highest positive rate of HPV was observed in those aged > 50 years (59.43%), followed by those aged ≤ 20 years (56.47%), showcasing a “U‐shaped” distribution across various age groups. The positive rates of syphilis and HSV‐2 exhibited an upward trend with increasing age, with visitors aged > 50 years had the highest positive rates, marked as 30.80% and 38.52%, respectively. UU and CT showed their highest positive rates in visitors aged ≤ 20 years, noted as 42.34% and 26.79%, respectively. Visitors aged > 50 years demonstrated the highest overall positive rate of STIs at 58.05%. Multiple infections exhibited the highest positive rate in visitors aged ≤ 20 years(8.05%). However, there was no significant difference in the positive rates of HIV, NG, and MG among symptomatic male visitors across different age groups (Table 2).

**Table 2:** Detection rates of different STIs in different ages of male.

		≤20	21−30	31−40	41−50	>50	*X* ^2^	*p*
Symptomatic	HPV	56.47% 48/85	45.75% 484/1058	46.79% 445/951	43.7% 163/373	59.43% 167/281	22.43	<.001
	HIV	0.51% 2/390	1.12% 39/3477	0.83% 28/3390	1.05% 19/1804	0.52% 9/1718	5.98	.201
	Syphilis	17.20% 75/436	17.47% 681/3898	14.71% 554/3766	19.71% 406/2060	30.80% 643/2088	238.261	<.001
	HSV‐2	7.55% 12/159	12.13% 66/1369	20.22% 308/1523	31.29% 266/850	38.52% 292/758	259.434	<.001
	UU	42.34% 47/111	29.45% 245/832	27.06% 273/1009	31.80% 166/522	27.11% 106/391	14.07	.007
	CT	26.79% 30/112	13.07% 103/788	10.42% 101/969	13.68% 68/497	10.55% 40/379	26.973	<.001
	NG	18.81% 19/101	13.55% 103/760	12.02% 111/923	13.29% 63/474	11.35% 42/370	4.921	.296
	MG	10.28% 11/107	9.59% 75/782	7.42% 71/957	7.24% 36/497	6.12% 23/376	6.109	.191
	Overall	38.43% 191/497	35.82% 1640/4578	36.35% 1611/4432	41.82% 1009/2413	58.05% 1146/1974	328.598	<.001
	Multiple infection	8.05% 40/497	4.61% 211/4578	5.19% 230/4432	6.05% 146/2413	7.40% 146/1974	28.667	<.001
Asymptomatic	HPV	36.67% 11/30	32.16% 136/423	33.64% 146/434	35.63% 62/174	54.95% 50/91	17.792	.001
	HIV	0% 0/117	1.74% 15/864	1.57% 12/766	0.79% 3/378	0.60% 2/331	4.177	.352 (F)
	Syphilis	12.00% 15/125	10.25% 89/868	14.56% 112/769	25.96% 101/389	49.32% 181/367	278.808	<.001
	HSV‐2	7.41% 2/27	5.71% 18/315	13.96% 43/308	25.63% 41/160	32.53% 27/83	57.935	<.001
	UU	35.29% 12/34	22.96% 73/318	28.85% 103/357	25.87% 37/143	27.06% 23/85	4.46	.347
	CT	10.81% 4/37	5.67% 20/353	3.71% 14/377	4.29% 6/140	3.70% 3/81	4.809	.307
	NG	0% 0/34	4.18% 14/335	3.29% 12/365	3.76% 5/133	1.33% 1/75	1.876	.744 (F)
	MG	12.50% 3/24	3.07% 8/261	5.21% 15/288	1.74% 2/115	0% 0/64	9.595	.033 (F)
	Overall	25.34% 37/146	28.16% 323/1147	35.65% 384/1077	41.45% 223/538	57.76% 268/464	138.529	<.001
	Multiple infection	4.79% 7/146	3.31% 38/1147	6.04% 65/1077	5.39% 29/538	3.66% 17/464	11.025	.026

Abbreviations: CT, chlamydia trachomatis; F, The p values were calculated using Fisher's exact test; HIV, human immunodeficiency virus; HPV, human papillomavirus; HSV‐2, herpes simplex virus type 2; MG, Mycoplasma genitalium; NG, neisseria gonorrhoeae; STIs, sexually transmitted infections; UU, Ureaplasma urealyticum.

Among asymptomatic male visitors, the positive rate of HPV, syphilis, and HSV‐2 in those aged ≤ 20 years was higher than that in those aged 21‐30 years, and then presented an upward trend after 21−30 years with the increase of age. Visitors aged＞50 years had the highest positive rate of HPV, syphilis, and HSV‐2, which were 54.95%, 49.32% and 32.53%, respectively. The highest positive rate of MG was observed in visitors aged ≤ 20 years, which was 12.50%. There was no significant difference in the positive rate of HIV, UU, CT, and NG among male visitors of different age groups. The highest positive rate of multiple infections was 6.04%, which was observed in visitors aged 31‐40 years (Table 2).

In symptomatic female visitors, the highest positive rates for HPV, UU, CT, NG, MG, and multiple infections were observed in visitors aged ≤ 20 years, with rates of 72.38%, 64.71%, 17.65%, 15.79%, 11.48%, and 11.04%, respectively. Similarly, the positive rates of syphilis and HSV‐2 increased with age, with aged > 50 years having the highest positive rates, marked as 49.43% and 69.83%, respectively. The highest positive rate for Candida, at 40.54%, was observed in visitors aged 21−30 years. While there was no significant difference in the overall positive rate of STIs among symptomatic female visitors of different age groups, the overall positive rate of STIs exceeded 50% at all ages (Table 3).

**Table 3:** Detection rates of different STIs in different ages of female.

		≤20	21−30	31−40	41−50	>50	*X* ^2^	*p*
Symptomatic	HPV	72.38% 131/181	58.15% 635/1092	49.75% 299/601	53.33% 168/315	59.47% 179/301	33.269	<.001
	HIV	0% 0/231	0% 0/1058	0% 0/722	0.37% 2/535	0.16% 1/61	/	/
	Syphilis	27.68% 93/336	33.35% 528/1583	33.33% 352/1056	42.69% 365/855	49.43% 565/1143	110.387	<.001
	HSV‐2	26.56% 17/64	39.80% 158/397	55.14% 193/350	60.36% 166/275	69.83% 169/242	79.883	<.001
	Candida	36.84% 42/114	40.54% 242/597	35.64% 144/404	34.12% 87/255	11.17% 20/179	53.226	<.001
	UU	64.71% 44/68	60.48% 228/377	57.63% 151/262	53.63% 96/179	44.59% 70/157	14.091	.007
	CT	17.65% 12/68	12.13% 42/346	6.53% 16/245	7.88% 13/165	3.27% 5/135	18.572	.001
	NG	15.79% 12/76	6.91% 26/376	7.91% 20/253	2.96% 5/169	4.38% 7/160	15.669	.003
	MG	11.48% 7/61	10.60% 32/302	7.11% 15/211	4.70% 7/149	3.62% 5/138	10.047	.04
	Overall	57.23% 285/498	59.09% 1567/2652	56.95% 996/1749	61.30% 795/1297	60.85% 920/1512	8.546	.074
	Multiple infection	11.04% 55/498	8.94% 237/2652	8.86% 155/1749	7.48% 97/1297	5.29% 80/1512	26.37	<.001
Asymptomatic	HPV	68.75% 22/32	46.85% 119/254	30.25% 49/162	25.30% 21/83	43.75% 21/48	30.143	<.001
	HIV	0% 0/111	0% 0/463	0% 0/319	0.47% 1/213	0% 0/208	/	/
	Syphilis	18.40% 23/125	19.14% 93/486	27.56% 97/352	37.45% 88/235	57.56% 137/238	127.261	<.001
	HSV‐2	31.25% 10/32	30.14% 44/146	45.71% 48/105	41.54% 27/65	34.88% 15/43	7.491	.122
	Candida	22.73% 5/22	24.14% 28/116	15.15% 10/66	10.26% 4/39	5.26% 1/19	7.211	.125
	UU	71.11% 32/45	63.24% 117/185	60.66% 74/122	65.75% 48/73	32.35% 11/34	14.777	.005
	CT	32.00% 16/50	14.16% 31/219	8.33% 12/144	6.85% 5/73	0% 0/34	24.445	<.001 (F)
	NG	31.37% 16/51	5.60% 13/232	5.92% 9/152	1.37% 1/73	2.78% 1/36	49.635	<.001
	MG	16.67% 6/36	14.65% 23/157	8.65% 9/104	3.17% 2/63	0% 0/27	11.868	.014 (F)
	Overall	53.38% 79/148	50.28% 354/704	49.59% 244/492	53.59% 164/306	57.86% 173/299	6.509	.164
	Multiple infection	20.95% 31/148	11.22% 79/704	9.96% 49/492	8.17% 25/306	4.01% 12/299	33.74	<.001

Abbreviations: CT, chlamydia trachomatis; F, The p Values were calculated using Fisher's exact test; HIV, human immunodeficiency virus; HPV, human papillomavirus; HSV‐2, herpes simplex virus type 2; MG, mycoplasma genitalium; NG, Neisseria gonorrhoeae; STIs, sexually transmitted infections; UU, ureaplasma urealyticum.

Similar to asymptomatic female visitors, the highest positive rates for HPV, UU, CT, NG, MG, and multiple infections were observed in those aged ≤ 20 years. The highest positive rate for syphilis occurred in visitors aged > 50 years. There were no significant differences in the positive rates of HSV‐2 and Candida, as well as in the overall positive rate, among different age groups. Unexpectedly, the positive rates of UU, CT, NG, and MG in asymptomatic visitors were higher than those in symptomatic visitors, particularly among visitors with lower ages (such as UU in visitors aged ≤ 50 years, CT in visitors aged ≤ 40 years, NG in visitors aged ≤ 20 years, and MG in visitors aged ≤ 40 years) (Table 3).

### 3.4 Influence of COVID‐19 on STIs

In early 2020, COVID‐19 emerged as a global pandemic. In response, Hangzhou, China, enforced a national epidemic prevention and control policy from January 25, 2020, to December 19, 2022. In this study, visitors were categorized based on the timeline of the COVID‐19 pandemic: before the pandemic (January 1, 2019, to January 25, 2020), during the pandemic (January 26, 2020, to December 19, 2022), and after the pandemic (December 20, 2022, to November 20, 2023). There were a total of 27,283 visitors, with 6439 people visiting before the pandemic, 15,968 people visiting during the pandemic, and 4876 people visiting after the pandemic. We then calculated the differences in the positive rates of STIs before, during, and after the COVID‐19 pandemic and also analyzed the impact of the pandemic on STIs. Results indicated that the positive rates of HPV, HIV, syphilis, and HSV‐2, as well as the overall positive rate of STIs in symptomatic visitors, were lower during the COVID‐19 pandemic compared to periods before and after the pandemic. Conversely, the positive rates of Candida, UU, CT, NG, and multiple infections in symptomatic visitors increased gradually before, during, and after the COVID‐19 pandemic. No significant change was observed in the positive rate of MG in symptomatic visitors. Among asymptomatic visitors, HPV exhibited the lowest positive rate during the COVID‐19 pandemic, while the highest positive rates for NG and multiple infections were observed in asymptomatic visitors during this period. The positive rates of syphilis, UU, and NG in asymptomatic visitors demonstrated an increasing trend before, during, and after the COVID‐19 pandemic. However, there was no significant difference in the positive rates of HIV, HSV, Candida, MG, and the overall positive rate of STIs in asymptomatic visitors before, during, and after the COVID‐19 pandemic (Table 4).

**Table 4:** Detection rates of different STIs before, during and after the COVID‐19 pandemic.

		Before the pandemic	During the pandemic	After the pandemic	*X* ^2^	*p*
Symptomatic	HPV	68.36% (806/1179)	45.78% (1442/3150)	51.82% (471/909)	175.303	<.001
	HIV	0.65% (20/3058)	0.63% (55/8705)	1.15% (25/2178)	6.734	.034
	Syphilis	27.21% (1019/3745)	23.79% (2462/10347)	25.10% (781/3111)	17.431	<.001
	HSV‐2	29.22% (521/1783)	27.96% (1013/3623)	36.66% (213/581)	9.598	.008
	Candida	37.71% (155/411)	35.19% (303/861)	26.42% (79/299)	10.672	.005
	UU	28.74% (121/421)	36.13% (966/2674)	41.70% (339/813)	20.574	<.001
	CT	7.75% (32/413)	11.75% (296/2519)	12.91% (102/790)	7.374	.025
	NG	6.28% (30/478)	10.70% (256/2393)	15.42% (122/791)	26.555	<.001
	MG	8.48% (33/389)	7.76% (187/2411)	7.95% (62/780)	0.251	.882
	Overall	50.93% (2449/4809)	44.46% (5904/13280)	46.66% (1807/2873)	59.697	<.001
	Multiple infection	5.24% (252/4809)	6.36% (844/13280)	7.77% (301/3873)	23.084	<.001
Asymptomatic	HPV	46.98% (249/530)	30.47% (245/804)	33.73% (143/424)	39.1	<.001
	HIV	0.78% (9/1147)	0.99% (20/2026)	0.67% (4/597)	0.691	.708
	Syphilis	20.45% (244/1193)	24.51% (517/2109)	26.84% (175/652)	11.293	.004
	HSV‐2	22.58% (84/372)	20.55% (158/769)	23.08% (33/143)	0.88	.644
	Candida	15.15% (10/66)	20.53% (31/151)	15.56% (7/45)	1.165	.558
	UU	34.90% (119/341)	42.25% (300/710)	43.59% (119/273)	6.413	.041
	CT	4.20% (14/333)	10.91% (91/834)	2.23% (6/269)	29.045	<.001
	NG	1.41% (5/354)	5.55% (44/793)	8.58% (23/268)	17.026	<.001
	MG	4.84% (6/124)	7.41% (50/675)	4.58% (12/262)	3.092	.213
	Overall	40.92% (667/1630)	43.19% (1161/2688)	42.67% (428/1003)	2.182	.336
	Multiple infection	3.80% (62/1630)	8.41% (226/2688)	6.48% (65/1003)	34.772	<.001

Abbreviations: CT, chlamydia trachomatis; HIV, human immunodeficiency virus; HPV, human papillomavirus; HSV‐2, herpes simplex virus type 2; MG, Mycoplasma genitalium; NG, Neisseria gonorrhoeae; STIs, sexually transmitted infections; UU, ureaplasma urealyticum.

## 4 DISCUSSION

People living with STIs often face barriers to seeking medical treatment, including mild symptoms, asymptomatic conditions, feelings of shame, and economic constraints. Additionally, some developing countries lack effective diagnostic facilities, resulting in a dearth of epidemiological data on STIs. In this comprehensive study conducted over 5 years, 27,283 first time visitors were enrolled in a STIs clinic in a major Chinese city. The prevalence of STIs and multiple infections was analyzed among those visitors of different genders, ages, and symptoms. Our study also assessed the impact of the COVID‐19 pandemic on STIs. Results revealed that symptomatic male and female visitors showed overall STI‐positive rates of 39.27% and 59.20%, respectively. The top three pathogens in both genders were HPV, UU, and HSV‐2. Among asymptomatic visitors, the total STI‐positive rate was 36.63% in males and 52.03% in females. Age‐stratified analysis revealed higher STI rates in visitors ≤ 20 or >50 years, regardless of gender and symptoms. During the COVID‐19 pandemic, symptomatic visitors showed lower positive rates for HPV, HIV, syphilis, and HSV‐2, while Candida, UU, CT, NG, and multiple infections increased. Among asymptomatic visitors, HPV had the lowest positive rate, while NG and multiple infections increased during the pandemic.

HPV stands as the most prevalent STI globally, with a high likelihood of exposure for most sexually active individuals. Regional and central variations in HPV prevalence exist, with an estimated average infection rate of approximately 11.7% among females with normal cervical cells. Sub‐Saharan Africa, Eastern Europe, and Latin America demonstrate the highest rates, while West Asia exhibits the lowest.^16^ Notably, the prevalence of HPV infections in male genitalia varies significantly across countries such as China, Brazil, Mexico, and the United States, ranging from 2% to 31%.^4^, ^17^ Consistent with global patterns, we also identified HPV as the pathogen with the highest positive rate. The positive rate for HPV was 47.56% and 56.71% among symptomatic male and female visitors, and 35.16% and 40.07% among asymptomatic male and female visitors, respectively. While many HPV infections are asymptomatic and self‐limiting, persistent infections can lead to serious health risks such as cervical cancer, anal cancer.^18^ HPV vaccination has proven effective in preventing 70%−90% of HPV‐related diseases, including anogenital warts and HPV‐related cancers.^17^ Currently, there are four commercial HPV vaccines available: Gardasil (Merck, 2006), Ceravrix (GSK, 2007), Gardasil 9 (Merck, 2014), and Cecolin (Innovax, 2021).^19^

UU is a prevalent pathogen in the reproductive tract, capable of invading the cell membrane. In asymptomatic female visitors, the separation rate of UU in the reproductive tract ranges from 40% to 80%.^20^ In the present study, the positive rate of UU was 29.21% and 26.47% among symptomatic and asymptomatic male visitors, respectively. Among symptomatic and asymptomatic female visitors, the positive rates were 56.47% and 61.44%, respectively. The positive detection of UU in the vagina has been associated with obstetric complications, including conditions related to premature and low birth weight newborns, as well as some neonatal diseases.^21^ However, the routine detection, high asymptomatic colonization rate of UU, and limited evidence on its pathogenic effects create uncertainty among clinicians regarding the necessity of treating people living with UU. Currently, there is no conclusive evidence supporting the routine detection and treatment of UU in adult males and nonpregnant females.^22^ Drug‐resistant pathogens may be identified during the extensive detection of UU and corresponding antimicrobial treatment, and may bring huge economic costs to society and individuals.

Globally, as many as 491.5 million individuals are infected with HSV‐2.^23^ In our presented results, the positive rate of HSV‐2 in symptomatic and asymptomatic visitors was 29.18% and 21.42%, respectively. The mainstay of therapy for HSV‐2 infection is symptomatic treatment. As of now, there is no conclusive evidence demonstrating that screening for HSV‐2 in asymptomatic individuals can effectively prevent the sequelae and spread of this disease. Consequently, screening for HSV‐2 in asymptomatic individuals, including pregnant individuals, is not recommended. The potential for false positive results, increased patient anxiety, and unnecessary risks associated with antiviral drugs outweigh the benefits of screening.^24^, ^25^

The positive rate of multiple infections of STIs varies significantly across different regions globally, such as 47% in Korea,^26^ 40% in India,^27^ 21% in Australia,^28^ and 7% in the Netherlands.^29^ In this study, the positive rate of multiple infections among symptomatic male and female visitors and asymptomatic male and female visitors was 5.42%, 8.10%, 4.63%, and 10.06%, respectively. Divergent STI patterns and varying definitions of multiple infections contribute to substantial differences in the reported rates of multiple infections across different studies. For instance, a Korean study encompassed 12 pathogens, reporting a multiple infections rate of 47%.^26^ In contrast, a study in the Netherlands calculated a lower multiple infections rate of 7% based on the attack/incidence rate.^29^

Regional differences in STIs and multiple infections may stem from various factors. Genetic variations, immune responses, individual lifestyles, and sanitary conditions can influence the susceptibility and persistence of STIs in diverse regions. Additionally, variations in medical resources, screening coverage, and diagnostic criteria may impact the detection levels and data quality of STIs. Cultural backgrounds, religious beliefs, and social concepts also contribute to regional disparities in awareness and acceptance of STI‐related knowledge. This underscores the importance of exploring the prevalence of STIs in different regions.

To identify differences in the positive rates of STIs among different age groups, visitors were stratified based on their age. The results revealed that the HPV positive rates are higher in visitors aged ≤20 years and >50 years compared to middle‐aged individuals, displaying a U‐shaped distribution, which may be influenced by several key factors related to the natural history of the infection, immunological responses, and behavioral patterns across different age groups.

Evolving societal norms and changing ideologies among young individuals, embracing more open notions of sexuality, contribute to the increased risk. However, the dissemination of health knowledge has not kept pace with these changes, leading to a lack of awareness or misunderstanding about the spread of STIs among many teenagers. The psychological need for affirmation in social groups further prompts teenagers to engage in risky sexual behavior. In addition, younger individuals' developing genital tissue and less previous exposure to HPV, and thus lower levels of natural immunity compared to older age groups, make adolescents more susceptible to infection. Similarly, the positive rate of UU, CT, NG, and MG were highest among visitors < 20 years old. This aligns with previous findings that highlight the heightened risk of STIs in young individuals. Adolescents, in particular, are 2−3 times more likely to contract STIs than adults, with the highest prevalence observed in individuals aged 15−19 years.^30^, ^31^

The rise in infection rates among the elderly can be attributed to several factors. First，older adults who did not benefit from HPV vaccination programs introduced in more recent decades remain susceptible to HPV infections. Second, HPV can persist in a latent state and potentially reactivate later in life, contributing to a resurgence in prevalence among older adults. Third: biological aging can exert certain impacts, as the decreasing immunity and hormone levels with age may render females, particularly after menopause, more susceptible to STIs due to anatomical changes in the genital tract. The narrowing and shortening of the vagina, reduced lubrication, and imbalances in vaginal flora contribute to heightened vulnerability.^32^ Moreover, the longer time required to arouse sexual desire in males may affect sexual health.^31^ Fourth, older adults, especially those who are divorced or widowed, may establish new sexual relationships, increasing their risk of acquiring HPV. Lastly, the lack of training for health care professionals to address STIs in elderly patientvisitors, influenced by stereotypes about aging and sexuality, may lead clinicians to avoid proactively discussing sexual health during health care service.^33^

The observed “infection gap” corresponds to the middle‐aged cohort, where lower infection rates are seen. This gap can be explained by the fact that they were exposed to HPV earlier in life and their immune systems may have developed partial immunity or cleared the infection, resulting in a lower prevalence. In addition, this age group tends to have more stable, monogamous relationships, reducing the likelihood of new HPV infections. They also had higher awareness and utilization of health interventions such as HPV vaccination and regular screening, which helped reduce infection rates in this cohort.

The “U‐shaped” curve in HPV prevalence highlights the complex interplay between age‐specific sexual behavior, immunological factors, and historical changes in HPV vaccination coverage. Understanding this pattern is crucial for effectively targeting public health interventions at high‐risk groups. Strengthening health education and public awareness, especially for adolescents and the elderly, can enhance their understanding of STI prevention. Preventive measures such as condom use, encouraging regular screening and early detection, and promoting HPV vaccination in age‐appropriate populations can help reduce incidence rates, mitigate adverse obstetric outcomes, and ultimately improve overall quality of life.

The COVID‐19 pandemic triggered profound social and economic shifts, impacting access to sexual health services.^34^ Consequently, it is of high value to explore the pandemic's influence on STIs. Our outcomes revealed a lower positive rate of HPV, HIV, syphilis, and HSV‐2, as well as the overall positive rate of STIs in symptomatic visitors during the COVID‐19 pandemic compared to periods before and after. Several factors contributed to this trend. Firstly, pandemic‐related measures such as stay‐at‐home orders, increased social distancing, and people who seek medical treatment in hospital and other public activities need to provide a negative COVID‐19 test report within the last 3 days which is aimed at ensuring public health safety and reducing the risk of the spread of the epidemic. However, it also reduces people's social activities, and reduces the spread of STIs in the population. Additionally, the closure of entertainment venues during the epidemic has positively affected STIs prevalence by reducing sexual activity, including fewer partners, extramarital encounters, and decreased sexual frequency. These behavioral changes, driven by measures like stay‐at‐home orders, social distancing, and health codes, contributed to a decline in STIs transmission. However, as pandemic‐related policies were lifted in the later stages, sexual activity increased, leading to a resurgence in STI prevalence, with reports suggesting a return to pre‐pandemic levels by June 2020.^35^, ^36^

Paradoxically, the positive rates of Candida, UU, CT, NG, and multiple infections in symptomatic visitors increased gradually before, during, and after the COVID‐19 pandemic. NG and multiple infections exhibited the highest positive rates in asymptomatic visitors during the COVID‐19 pandemic, while syphilis, UU, and NG demonstrated an increasing positive rate before, during, and after the pandemic. These findings suggest that the COVID‐19 pandemic had broad and varied impacts on STI transmission. Model‐based studies propose that the interruption of clinical and preventive services could lead to an increase in infection rates, offsetting the effects of reduced sexual partners. Increased STI infection rates during the pandemic, particularly for CT, can be attributed to the disruption of clinical services.^37^ Other research supports these findings, confirming that interruptions in health care services for STIs during the pandemic can contribute to a small increase in CT infection rates.^38^

In conclusion, the pandemic's impact on STI rates was not uniform across all STIs. While certain infections decreased due to reduced sexual activity, others may have increased due to the diminished availability of timely medical care and screening. Social factors, such as limited access to health care services, reluctance to seek medical help due to fear of COVID‐19, and the redeployment of health care resources to combat the pandemic, likely played a significant role in these trends. These results underscore the critical importance of consistent prevention, health care, and nursing measures in controlling the spread and prevalence of STIs, even during public health emergencies. To better understand the pandemic's impact on STI transmission, further research should focus on disentangling these complex interactions between social behavior, health care access, and STI epidemiology.

Nevertheless, there are also some limitations in this study that need to be acknowledged. First, the data were collected from a single clinic, which may limit the generalizability of the findings to broader populations. The specific demographic and geographic characteristics of the clinic's visitors may not be representative of other regions or populations, which could influence the applicability of the results. Second, the presence or absence of clinical symptoms of partial visitors is determined based on their self‐reported symptoms. It's important to note that visitors may not always report their condition accurately to clinicians, which could lead to underreporting of asymptomatic cases. Additionally, diagnoses based on “health examination” by clinicians might lead to misclassification as asymptomatic visitors in some individuals, introducing potential bias. Furthermore, economic considerations and individual conditions may affect the extent of testing, leading to variations in the number and types of pathogen tests each visitor receives. As a result, not all visitors are tested for all pathogens, and some may only receive partial testing, potentially influencing the overall positive rate of STIs and the observed rate of multiple infections, making it crucial to interpret the results with these considerations in mind.

## 5 CONCLUSION

The findings of this study emphasize the high prevalence of STIs among the first‐time visitors of STIs clinic, regardless of symptom presence. This highlights the critical need for routine STI screening, especially for HPV, when economic conditions allow. Health education programs should prioritize raising awareness and promoting preventive measures, such as condom use and HPV vaccination, particularly among high‐risk age groups. The COVID‐19 pandemic, while reducing sexual activity, may have inadvertently led to higher STI rates due to decreased screening and disruptions in health care services. These results emphasize the need for resilient health care systems that can continue to address STI prevention and treatment even during public health crises.

## AUTHOR CONTRIBUTIONS

**Jiyun Tian**: Investigation, methodology, writing—original draft, writing—review and editing. **Shi Chen**: Data curation. **Xinzheng Li**: Data curation, investigation. **Yong Teng**: Resources. **Baobing Chen**: Conceptualization, methodology.

## CONFLICT OF INTEREST STATEMENT

The authors declare no conflict of interest.

## ETHICS STATEMENT

This study was approved by the Ethics committee of Hangzhou Third People's Hospital. The approval number is 2023KA096. Since this was a retrospective study, the Ethics Committee have waived informed consent.
